# Normative reference values and predicting factors of handgrip strength for dominant and non-dominant hands among healthy Malay adults in Malaysia

**DOI:** 10.1186/s12891-023-06181-8

**Published:** 2023-01-28

**Authors:** Mohd Hasni Jaafar, Rosnah Ismail, Noor Hassim Ismail, Zaleha Md Isa, Azmi Mohd Tamil, Nafiza Mat Nasir, Kien Keat Ng, Nurul Hafiza Ab Razak, Najihah Zainol Abidin, Khairul Hazdi Yusof

**Affiliations:** 1 Department of Community Health, Faculty of Medicine, UKM Medical Centre, Universiti Kebangsaan Malaysia, Cheras, Kuala Lumpur, Malaysia; 2 Department of Primary Care Medicine, Faculty of Medicine, Universiti Teknologi MARA Sungai Buloh, Sungai Buloh, Selangor Malaysia; 3 Faculty of Medicine & Defence Health, National Defence University of Malaysia, Kem Perdana Sungai Besi, Sungai Besi, Kuala Lumpur, Malaysia; 4 Department of Diagnostic & Allied Health Science, Faculty of Health and Life Sciences, Management & Science University, Shah Alam, Selangor Malaysia

**Keywords:** Handgrip strength (HGS), Reference values, Adult, Malaysia, Health status

## Abstract

**Introduction:**

Handgrip strength (HGS) measures the maximum voluntary force of the hand, which has been used to assess individual health status indirectly. Although several factors related to HGS have been identified, studies among adults in Malaysia are lacking. This study aimed to provide the normative reference HGS values and determine its predictor factors among healthy adults of Malay ethnic in Malaysia.

**Methodology:**

This study was a part of the Prospective Urban Rural Epidemiology (PURE) study carried out among adults aged between 35 to 70 years old residing in urban and rural Malaysian communities. A standardised questionnaire was used to assess the socio-demographic information and physical activity level of respondents who provided written informed consent to participate in this study. HGS was measured using Jamar’s dynamometer. A total of 3,446 healthy adults of Malay ethnic were included in this study. Descriptive data were used to derive the normative reference values for HGS using means and standard deviations stratified by age and gender. The predictors of HGS were determined using a general linear model (GLM).

**Results:**

Mean HGS ranged from 38.48 (± 9.40) kg for the dominant hand of men aged 35–40 years to 16.53 (± 5.69) kg for the non-dominant hand of women aged 61–70 years. The ANOVA indicated that there was a significant descending trend of HGS as age increased for both genders (*p* < 0.05). The ANOVA also revealed that participants working in blue- or white-collar jobs had higher HGS than their counterparts who are homemakers (*p* < 0.05). The GLM shows that, age, occupation type, socio-economic status (SES), physical activity level and BMI significantly predicted dominant HGS among male and meanwhile, only age, SES and BMI significantly predicted dominant HGS among female.

**Conclusion:**

HGS normative values provided herein can serve as a guide for interpreting HGS measurements obtained from healthy Asian adults especially Malay ethnic. The clinicians and researcher can use the established HGS reference values as comparison in their patients or participants. Furthermore, during a rehabilitation process, the clinicians and researchers could use the normal score ranges to document the progress of HGS and provide feedback to the patients. Further study with prospective study design is needed to determine the causal effect association of the predictors and HGS.

## Introduction

Handgrip strength (HGS) is mainly considered as part of standard physical examination compared to vital sign parameters in a clinic setting. HGS measures the highest voluntary strength of the hand and is normally determined using a dynamometer [1]. Its ease of measurement with minimal training may provide a risk-stratifying screening tool for muscular strength and neuromuscular functioning and indirectly for cardiovascular or pulmonary conditions, nutritional status, or testing of frailty and sarcopenia [1–3]. It is useful for assessing upper extremities disorders and post-injury evaluation as well as an athletic performance index [4]. In addition, HGS weakness also has prognostic ability to determine health risks, including all-cause death and cardiovascular mortality [2].

The lack of a worldwide applicable reference range for HGS may explain why it is rarely adopted in a clinical and epidemiological setting. Although previous peer-reviewed studies provided such reference values, the studies mostly used small convenient samples [3]. Furthermore, many studies were conducted to determine the reference values for Western high-income countries, such as the United States, Great Britain, Germany, Spain, Australia and Canada [2, 3]. Previous studies conducted in Korea, Taiwan, Japan and Malaysia have revealed that HGS among the Eastern population were much lower by 1.5 times compared to the Western population [2–5]. Therefore, Western reference values are not suitable for the Eastern population and additional attention is needed to identify various factors that influence HGS to determine representative values.

HGS is influenced by many factors, such as age, gender, height, weight, hand dominance, occupation, physical activity level and geographical areas [4, 6]. Previous studies have found that HGS has a nonlinear relationship with age, usually peaking during the 4^th^ decade of life and then declining gradually [6]. HGS also increases linearly with height and weight and is notably higher in the dominant hand [4, 6].

Based on our literature search, only three previous studies have reported HGS reference values for the multi-ethnic Malaysian population [2, 4, 5]. However, to our knowledge, no study has focused on Malay ethnic individuals, who comprise a large urban and rural population. Hence, this study aimed to determine the normative reference HGS values and their predictors among healthy adults of Malay ethnic in Malaysia.

## Methodology

### Study design & participants

The design of the PURE study has been described elsewhere [7]. In short, PURE is a large-scale international cohort study that endeavours to establish the incidence, mortality and risk factors of non-communicable diseases among individuals from urban and rural communities in 21 countries, including Malaysia. This study has enrolled 15,792 Malaysian adults aged between 35 and 70 years at the baseline stage.

Purposive sampling was performed with the help of community leaders. Home visits were conducted, and all eligible individuals residing in the same household were invited to join the study. Only participants intend to continue stay at their current home for another at least 4 years and willing to participate in lengthy follow up study were included. Participants were excluded if HGS values were not recorded, their ethnicity was not Malay or had a history of diabetes, hypertension or CVDs. All participants were provided written informed consent prior to enrolment in this study. For illiterate participants, a written informed consent was obtained from legally authorized representative.

For quality control to ensure standardised methods of data collection, research assistants were trained with comprehensive operation manuals, videos and workshops. Data were transferred electronically to the project office and coordinating centre at PHRI for quality-control checks of the data. The protocol was approved by the Hamilton Health Sciences Research Ethics Board (PHRI), Research and Ethics Committee (UKM Medical Centre) and Research Ethics Committee (UiTM) (Project code: PHUM-2012–01).

### Procedures

Trained research assistants administered a validated standardised set of questionnaires to the participants. The questionnaire extracted self-reported demographic information, such as gender, age (rounded from date of birth), ethnicity (Malay or non-Malay), education level (none, primary, secondary or tertiary), occupation (blue-collar, white-collar or homemaker), marital status (single, married or separated), socio-economic status (low, middle or high) and health history (diabetes, hypertension and CVDs). Then, the physical activity level (low or high) of participants were elicited using the International Physical Activity Questionnaire (IPAQ).

HGS was measured using the Jamar dynamometer (Sammons Preston, Bolingbrook, IL, USA) according to a standardised protocol described previously [2]. The arm was positioned vertically to the body and the dynamometer was held with the elbow flexed to 90º. The participant was instructed to squeeze the device as hard as possible for 3 s. The measurement was repeated thrice at intervals of at least 30 s. The HGS data could be affected by the time duration over which a participant is required to activate the dynamometer, the learning effect and any encouragement given by the researchers. These issues were handled by giving each respondent a short time to familiarise himself with the dynamometer, a short hands-on practice and a few trials using the equipment. Three measurements were made from both hands of each participant. In this study, the maximum values obtained from each hand were used, namely dominant HGS, non-dominant HGS and highest HGS (either dominant or non-dominant HGS). Height and weight were measured using a portable height measuring scale stature meter and TANITA (BC-558 Ironman®) segmental body composition analyser. Body mass index (BMI) was derived by dividing weight by height squared.

### Statistical analysis

HGS reference values were computed and stratified by gender and age categories (35–40 years, 41–50 years, 51–60 years and 61–70 years). Then, t-test and one-way ANOVA were conducted to analyse the mean differences of HGS between the following variables: age, gender, marital status, education level, occupation, socio-economic status (SES) and physical activity level.

A general linear model (GLM) was computed to determine the predictors of the HGS. These predictors included age, marital status, occupation, SES, physical activity level, height and weight. Education level was excluded in the GLM due to multicollinearity with age. Assumptions of linearity, independence of errors, homoscedasticity and normality of residuals were met.

## Results

**Fig. 1:**
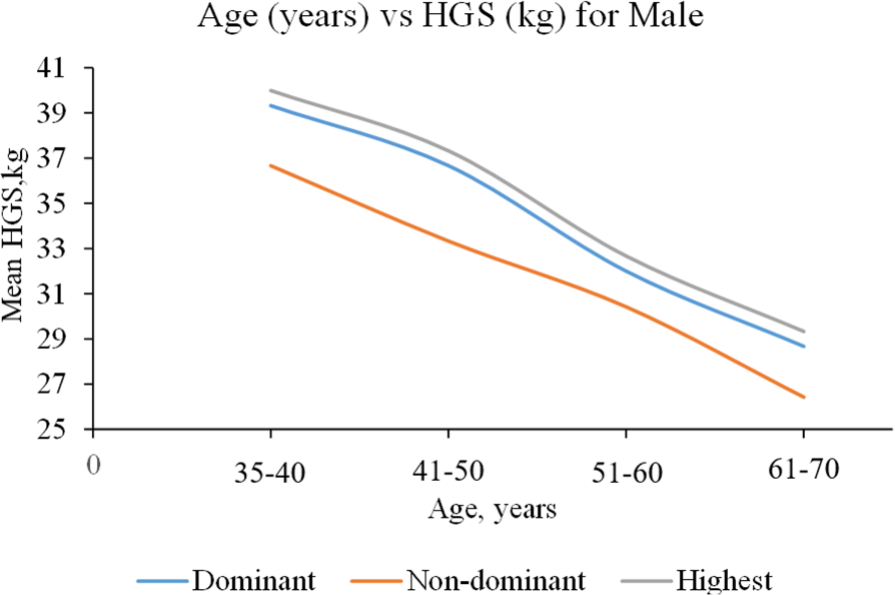
Mean HGS as a function of age (male participants)

**Fig. 2:**
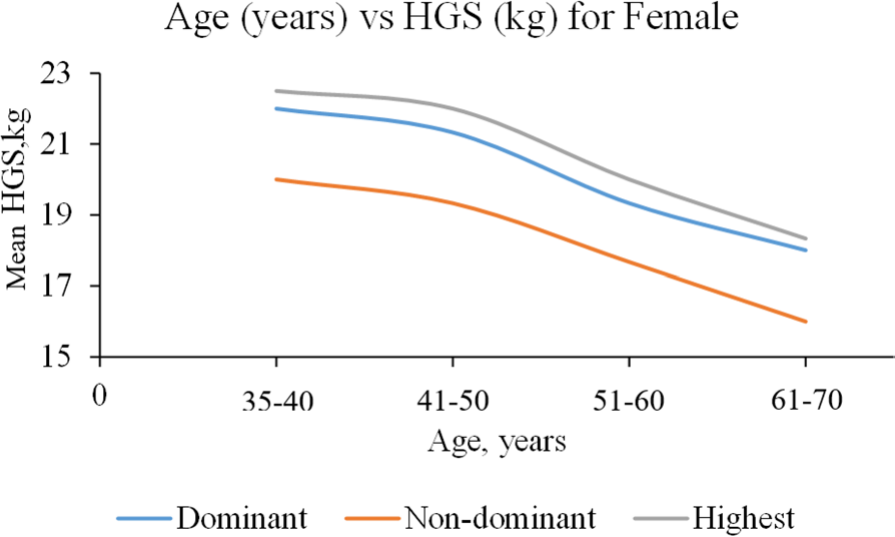
Mean HGS as a function of age (female participants)

A total of 3,446 healthy adults of Malay ethnic were included in this study. HGS ranges by age and gender are presented in Table 1. HGS among males exceeded HGS in females (Table 2). The HGS of the dominant hand also shows a higher value compared to the non-dominant hand. The highest HGS values were 39.43 (± 9.03) kg and 22.78 (± 5.36) kg for males and females, respectively, and both values were found in the age group of 35–40 years. Both genders showed progressive declines in HGS with increasing age (Figs. 1 and 2).

**Table 1:** Mean of HGS among respondents by age group

Age (year)	Male	HGS, kg		
	N	Dominant	Non-dominant	Highest
35–40	208	38.48 (9.40)	36.33 (8.52)	39.43 (9.03)
41–50	487	37.03 (8.27)	34.09 (7.77)	37.82 (7.97)
51–60	481	32.48 (7.88)	30.25 (7.65)	33.20 (7.74)
61–70	248	29.04 (7.72)	27.25 (7.55)	29.93 (7.43)
Age (year)	Female	HGS, kg		
	N	Dominant	Non-dominant	Highest
35–40	419	22.38 (5.54)	20.10 (5.27)	22.78 (5.36)
41–50	803	22.06 (5.97)	19.81 (5.66)	22.51 (5.83)
51–60	540	19.75 (5.30)	17.74 (5.07)	20.25 (5.13)
61–70	260	18.14 (5.90)	16.53 (5.69)	18.70 (5.84)

**Table 2:** Comparisons of handgrip strength measurements by demographic characteristics (*N* = 3446, male = 1424, female = 2022)

	Male	HGS, kg			Female	HGS, kg		
Variable	N	Dominant Mean (SD)	Non-dominant Mean (SD)	Highest Mean (SD)	N	Dominant Mean (SD)	Non-dominant Mean (SD)	Highest Mean (SD)
Age (year)								
35–40	208	**38.48 (9.40)**^**a**^	**36.33 (8.52)**^**b**^	**39.43 (9.03)**^**c**^	419	**22.38 (5.54)**^**a**^	**20.10 (5.27)**^**b**^	**22.78 (5.36)**^**c**^
41–50	487	**37.03 (8.27)**^**a**^	**34.09 (7.77)**^**b**^	**37.82 (7.97)**^**c**^	803	**22.06 (5.97)**^**a**^	**19.81 (5.66)**^**b**^	**22.51 (5.83)**^**c**^
51–60	481	**32.48 (7.88)**^**a**^	**30.25 (7.65)**^**b**^	**33.20 (7.74)**^**c**^	540	**19.75 (5.30)**^**a**^	**17.74 (5.07)**^**b**^	**20.25 (5.13)**^**c**^
61–70	248	**29.04 (7.72)**^**a**^	**27.25 (7.55)**^**b**^	**29.93 (7.43)**^**c**^	260	**18.14 (5.90)**^**a**^	**16.53 (5.69)**^**b**^	**18.70 (5.84)**^**c**^
Marital status								
Single	20	34.86 (8.96)	33.03 (6.95)	35.98 (7.89)	50	**21.31 (5.25)**^**a**^	**19.05 (4.22)**^**b**^	**21.79 (4.95)**^**c**^
Married	1372	34.46 (9.23)	32.05 (8.60)	35.27 (9.00)	1754	**21.28 (5.86)**^**a**^	**19.11 (5.61)**^**b**^	**21.74 (5.72)**^**c**^
Separated	27	32.48 (8.99)	29.32 (9.26)	32.75 (9.17)	213	**18.63 (5.77)**^**a**^	**17.12 (5.35)**^**b**^	**19.18 (5.59)**^**c**^
Education level								
None	65	**29.75 (11.26)**^**a**^	**28.38 (10.92)**^**b**^	**30.52 (10.98)**^**c**^	213	**17.92 (6.09)**^**a**^	**16.46 (6.10)**^**b**^	**18.70 (5.97)**^**c**^
Primary	414	**31.78 (8.08)**^**a**^	**29.77 (7.64)**^**b**^	**32.66 (7.75)**^**c**^	523	**20.34 (5.92)**^**a**^	**18.14 (5.73)**^**b**^	**20.76 (5.87)**^**c**^
Secondary	641	**35.71 (9.23)**^**a**^	**33.19 (8.68)**^**b**^	**36.48 (9.10)**^**c**^	943	**21.75 (5.66)**^**a**^	**19.53 (5.32)**^**b**^	**22.19 (5.45)**^**c**^
Tertiary	304	**36.30 (9.06)**^**a**^	**33.32 (8.29)**^**b**^	**37.09 (8.72)**^**c**^	342	**21.89 (5.63)**^**a**^	**19.81 (5.11)**^**b**^	**22.30 (5.50)**^**c**^
Occupation								
White collar	651	**34.85 (8.81)**^**a**^	**32.14 (8.23)**^**b**^	**35.55 (8.65)**^**c**^	586	**21.74 (5.54)**^**a**^	**19.59 (5.24)**^**b**^	**22.16 (5.41)**^**c**^
Blue collar	692	**34.60 (9.37)**^**a**^	**32.40 (8.73)**^**b**^	**35.48 (9.06)**^**c**^	197	**21.45 (6.62)**^**a**^	**19.70 (6.04)**^**b**^	**22.07 (6.28)**^**c**^
Homemaker	60	**28.58 (9.28)**^**a**^	**26.51 (7.99)**^**b**^	**29.37 (8.80)**^**c**^	1229	**20.56 (5.89)**^**a**^	**18.43 (5.63)**^**b**^	**21.03 (5.76)**^**c**^
SES								
Low	450	**33.00 (9.12)**^**a**^	31.29 (8.33)	**34.01 (8.63)**^**c**^	575	20.88 (6.14)	19.14 (5.83)	21.49 (5.93)
Medium	825	**35.20 (9.39)**^**a**^	32.43 (8.84)	**35.90 (9.30)**^**c**^	1215	21.07 (5.97)	18.74 (5.64)	21.47 (5.84)
High	149	**34.40 (7.92)**^**a**^	31.82 (7.83)	**35.17 (7.80)**^**c**^	232	20.98 (4.73)	19.10 (4.49)	21.43 (4.61)
Physical activity								
Low	440	**33.12 (9.18)**^**a**^	**31.09 (8.82)**^**b**^	**34.06 (9.03)**^**c**^	563	20.59 (5.84)	18.43 (5.47)	21.07 (5.72)
High	844	**34.99 (9.14)**^**a**^	**32.40 (8.42)**^**b**^	**35.76 (8.92)**^**c**^	1323	21.04 (5.82)	18.99 (5.49)	21.50 (5.64)

^a^ mean values are significantly different (*p* < 0.05) between variable group for dominant HGS

^b^ mean values are significantly different (*p* < 0.05) between variable group for non-dominant HGS

^c^ mean values are significantly different (*p* < 0.05) between variable group for highest HGS

Table 2 presents the socio-demographic characteristics of age, marital status, education level, occupation type, SES status and physical activity level stratified by gender. There was a significant descending trend of HGS as age increased for both genders (*p* < 0.05). Among females, the lowest HGS were observed in those who indicated they were separated compared to single and married (*p* < 0.05). However, no significant difference was observed in males based on marital status. Participants working in blue- or white-collar jobs had higher HGS than their counterparts who are homemakers (*p* < 0.05). Among males, a significant ascending trend of HGS was observed as SES status and physical activity level increased (*p* < 0.05). Meanwhile, no significant difference was observed in females considering the same demographic characteristics.

In the GLM, age, occupation type, SES status, physical activity level and BMI significantly predicted dominant HGS among male (Table 3). However only age, SES status and BMI significantly predicted dominant HGS among female. For non-dominant HGS among male, age, occupation type, physical activity level and BMI were significant predictors (Table 4). While age, occupation type, SES status, and BMI were significant predictors for non-dominant HGS among female. Table 5 shows that age, occupation type, SES status, physical activity level and BMI significantly predicted highest HGS among male. Whereas only age, SES status and BMI significantly predicted highest HGS among female. In general, participants who were older tended to have lower HGS. In contrast, blue-collar jobs, higher physical activity and greater BMI had higher HGS.

**Table 3:** GLM for dominant HGS

Gender	Predictor	R^2^	Beta (SE)	*p*-value	Effect size	F (df)
Male		0.18				30.83 (9)
	Age					
	35–40^a^					
	41–50		**-1.66 (0.69)**	**0.016**	**0.005**	
	51–60		**-5.84 (0.70)**	** < 0.001**	**0.053**	
	61–70		**-8.83 (0.80)**	** < 0.001**	**0.088**	
	Occupation					
	White collar		**4.53 (1.12)**	** < 0.001**	**0.013**	
	Blue collar		**4.91 (1.10)**	** < 0.001**	**0.015**	
	Homemaker^a^					
	SES					
	Low		0.63 (0.85)	0.456	-	
	Medium		**1.77 (0.76)**	**0.021**	**0.004**	
	High^a^					
	Physical activity					
	Low^a^					
	High		**1.50 (0.48)**	**0.002**	**0.008**	
	BMI		**0.26 (0.05)**	** < 0.001**	**0.019**	
Female		0.08				18.25 (9)
	Age					
	35–40^a^					
	41–50		-0.38 (0.35)	0.284	0.001	
	51–60		**-2.65 (0.38)**	** < 0.001**	**0.025**	
	61–70		**-4.03 (0.48)**	** < 0.001**	**0.037**	
	Occupation					
	White collar		0.51 (0.32)	0.109	0.001	
	Blue collar		0.57 (0.45)	0.206	0.001	
	Homemaker^a^					
	SES					
	Low		**0.99 (0.49)**	**0.042**	**0.002**	
	Medium		0.22 (0.44)	0.608	-	
	High^a^					
	Physical activity					
	Low^a^					
	High		0.26 (0.29)	0.362	-	
	BMI		**0.13 (0.02)**	** < 0.001**	**0.016**	

^a^reference group; 35–40 years, homemaker, high SES status, low physical activity

**Table 4:** GLM for non-dominant HGS

Gender	Predictor	R^2^	Beta (SE)	*p*-value	Effect size	F (df)
Male		0.17				27.59 (9)
	Age					
	35–40^a^					
	41–50		**-2.53 (0.66)**	** < 0.001**	**0.012**	
	51–60		**-6.04 (0.66)**	** < 0.001**	**0.062**	
	61–70		**-8.84 (0.77)**	** < 0.001**	**0.096**	
	Occupation					
	White collar		**3.99 (1.07)**	** < 0.001**	**0.011**	
	Blue collar		**4.89 (1.06)**	** < 0.001**	**0.017**	
	Homemaker^a^					
	SES					
	Low		1.04 (0.81)	0.199	0.001	
	Medium		1.35 (0.73)	0.063	0.003	
	High^a^					
	Physical activity					
	Low^a^					
	High		**0.96 (0.45)**	**0.035**	**0.004**	
	BMI		**0.23 (0.05)**	** < 0.001**	**0.016**	
Female		0.08				17.20 (9)
	Age					
	35–40^a^					
	41–50		**-0.45 (0.33)**	**0.175**	**0.001**	
	51–60		**-2.45 (0.36)**	** < 0.001**	**0.024**	
	61–70		**-3.44 (0.45)**	** < 0.001**	**0.031**	
	Occupation					
	White collar		**0.65 (0.30)**	**0.033**	**0.002**	
	Blue collar		**0.98 (0.42)**	**0.021**	**0.003**	
	Homemaker^a^					
	SES					
	Low		**0.99 (0.47)**	**0.032**	**0.002**	
	Medium		0.27 (0.41)	0.516	-	
	High^a^					
	Physical activity					
	Low^a^					
	High		0.40 (0.27)	0.138	-	
	BMI		**0.13 (0.02)**	** < 0.001**	**0.016**	

^a^reference group; 35–40 years, homemaker, high SES status, low physical activity

**Table 5:** GLM for highest HGS

Gender	Predictor	R^2^	Beta (SE)	*p*-value	Effect size	F (df)
Male		0.19				32.84 (9)
	Age					
	35–40^a^					
	41–50		**-1.90 (0.67)**	**0.005**	**0.006**	
	51–60		**-6.14 (0.67)**	** < 0.001**	**0.062**	
	61–70		**-9.02 (0.78)**	** < 0.001**	**0.096**	
	Occupation					
	White collar		**4.47 (1.09)**	** < 0.001**	**0.013**	
	Blue collar		**5.10 (1.07)**	** < 0.001**	**0.018**	
	Homemaker^a^					
	SES					
	Low		0.73 (0.82)	0.375	0.001	
	Medium		**1.59 (0.74)**	**0.031**	**0.004**	
	High^a^					
	Physical activity					
	Low^a^					
	High		**1.29 (0.46)**	**0.005**	**0.006**	
	BMI		**0.27 (0.05)**	** < 0.001**	**0.022**	
Female		0.08				18.98 (9)
	Age					
	35–40^a^					
	41–50		-0.36 (0.34)	0.298	0.001	
	51–60		**-2.58 (0.37)**	** < 0.001**	**0.025**	
	61–70		**-3.92 (0.46)**	** < 0.001**	**0.037**	
	Occupation					
	White collar		0.52 (0.31)	0.096	0.001	
	Blue collar		0.73 (0.44)	0.097	0.001	
	Homemaker^a^					
	SES					
	Low		**1.15 (0.48)**	**0.016**	**0.003**	
	Medium		0.17 (0.42)	0.690	-	
	High^a^					
	Physical activity					
	Low^a^					
	High		0.26 (0.28)	0.354	-	
	BMI		**0.14 (0.02)**	** < 0.001**	**0.018**	

^a^reference group; 35–40 years, homemaker, high SES status, low physical activity

## Discussion

This study aimed to provide normative reference values based on data obtained from healthy Malay ethnic adults in Malaysia. The highest HGS among Malay ethnic adults was from those aged 35–40 years using their dominant hand: 38.48 (± 9.40) kg for males and 22.38 (± 5.54) kg for females. These values were lower compared to the same age group of adults from the United States: 47.1 (± 11.9) and 28.0 (± 6.0) kg for males and females, respectively [3]. Similar trend were observed when HGS of this study were compared to the same age group of Iranian adults which has reported to have HGS of 53.0 (± 7.6) and 29.9 (± 7.5) kg for males and females, respectively [8]. A study done in Brazil also have reported higher HGS compared to this study, which were 46.9 (± 10.4) kg for males and 29.4 (± 6.4) kg for females [9]. The Korean adult population from the same age group also recorded higher HGS compared to this study: 46.0 (± 7.2) and 27.2 (± 4.6) kg for males and females, respectively [10].

This study has found that males had higher handgrip strength than females, which is similar to other previous studies [1, 2, 11]. This could be due to the greater muscle mass among males, which is 10% higher muscle mass compared to females on average [1, 12]. Moreover, Schorr et al. [12] suggested HGS differences between genders may be influenced by the differences in height and weight, as males are generally taller and heavier than females.

According to Jamir et al. [13] height and arm span are positively correlated, which means a taller person would have longer arms with a greater lever arm for force generation that results in an efficient amount of force compared to a shorter person [1]. Regarding weight, Moy et al. stated that greater body weight results in a higher muscle mass, which leads to higher HGS compared to those with lower body weight [1]. Furthermore, Rostamzadeh et al. [6] found that larger handbreadth and forearm circumference was related to higher HGS. However, both weight and muscle mass tend to decrease with older age [1, 6].

This study revealed support from previous findings that older participants tended to have lower HGS compared to younger participants [1, 2, 4, 11, 14]. The main reason for this result was the declining muscle mass among elders, which equals 10% of declining strength per decade starting at age 40 years [1, 11, 15, 16]. This is due to decreasing thickness of the anterior forearm muscles with ageing, which contributes to reduced HGS [17].

This study has revealed that lower SES status was related to higher HGS. This finding might be related to job demands among lower SES status occupations, for instance, blue-collar workers typically require more physical activity and strength in their daily work compared to other occupations [18]. This study has also found that being a blue-collar worker is a significant predictor of HGS. Similarly, a study conducted in Iran concluded that HGS among blue-collar workers (i.e. cleaner, gardener and driver) was 12% higher compared to the white-collar workers (clerk and administrative worker) [6]. This is mainly because repetitive movements in their routine daily work activities increase forearm muscle (flexor digitorum profundus and flexor pollicis longus) strength and muscle mass, which lead to increased HGS among blue-collar workers [6, 18, 19].

Furthermore, this study also found high physical activity was also a significant predictor of HGS among male in this study population. The findings are coherent with previous studies that reported higher physical activity was positively related to higher HGS [1, 20]. A review by Ramsey et al. [21] also reported that higher physical activity and lower sedentary behaviour were associated with higher HGS. HGS could be improved by physical activities that mainly target upper extremity exercises in which handgrip movements are performed, such as dumbbell lifting, push-ups and plank exercises [22]. Nordic walking also could improve HGS among elders by increasing muscular endurance and capacity, as the exercise includes a repetitive movement like a gripping task by holding the pole and executing the correct Nordic walking technique [22].

The main strength of this study is that it is able to provide the normative values for healthy Malay ethnic adults in Malaysia using a larger population survey in both urban and rural areas compared to other studies conducted in Malaysia. Furthermore, this study has provided cross-sectional data supported by the fitted predictors model. The limitation of this study is that only Malay ethnicity were included. Therefore, the normative value only can be applied to this population in Malaysia. However, a previous study conducted in Malaysia reported that HGS was not related to ethnicity but more related to age and anthropometric data [4].

## Conclusion

In conclusion, HGS for Asians is notably lower compared to the Western population. Therefore, the normative data provided in this study is beneficial for Asian medical and ergonomic research, as clinicians and researchers can compare the HGS values in individuals to the reference values established. Furthermore, they could document the progress of HGS and provide feedback during the rehabilitation process with the knowledge of normal score ranges for individuals from a similar Asian population. Older age was found to predict lower HGS, whereas the blue-collar jobs, higher physical activity and greater BMI predicted higher HGS. Further study with prospective study design is needed to determine the causal effect association of the predictors and HGS.

## Data Availability

The data that support the findings of this study are available from PHRI but restrictions apply to the availability of these data, which were used under license for the current study, and so are not publicly available. Data are however available from the corresponding author (Rosnah Ismail) upon reasonable request and with permission of PHRI.

## Abbreviations

HGS: Handgrip strength
PURE: Prospective urban and rural epidemiological study
CVDs: Cardiovascular diseases
SES: Socio-economic status
GLM: General linear model
